# Carbon Sequestration Potential in Stands under the Grain for Green Program in Southwest China

**DOI:** 10.1371/journal.pone.0150992

**Published:** 2016-03-09

**Authors:** Xiangang Chen, Yunjian Luo, Yongfeng Zhou, Mei Lu

**Affiliations:** 1 School of Environmental Science and Engineering, Southwest Forestry University, Kunming, Yunnan, China; 2 Department of Ecology, School of Horticulture and Plant Protection, Yangzhou University, Yangzhou, Jiangsu, China; Tennessee State University, UNITED STATES

## Abstract

The Grain for Green Program (GGP) is the largest afforestation and reforestation project in China in the early part of this century. To assess carbon sequestration in stands under the GGP in Southwest China, the carbon stocks and their annual changes in the GGP stands in the region were estimated based on the following information: (1) collected data on the annually planted area of each tree species under the GGP in Southwest China from 1999 to 2010; (2) development of empirical growth curves and corresponding carbon estimation models for each species growing in the GPP stands; and (3) parameters associated with the stands such as wood density, biomass expansion factor, carbon fraction and the change rate of soil organic carbon content. Two forest management scenarios were examined: scenario A, with no harvesting, and scenario B, with logging at the customary rotation followed by replanting. The results showed that by the years 2020, 2030, 2040, 2050 and 2060, the expected carbon storage of the GGP stands in Southwest China is 139.58 TgC, 177.50–207.55 TgC, 196.86–259.65 TgC, 240.45–290.62 TgC and 203.22–310.03 TgC (T = 10^12^), respectively. For the same years, the expected annual change in carbon stocks is 7.96 TgCyr^−1^, −7.95–5.95 TgCyr^−1^, −0.10–4.67 TgCyr^−1^, 4.31–2.24 TgCyr^−1^ and −0.02–1.75 TgCyr^−1^, respectively. This indicates that the stands significantly contribute to forest carbon sinks in this region. In 2060, the estimated carbon stocks in the seven major species of GGP stands in Southwest China are 4.16–13.01 TgC for *Pinus armandii*, 6.30–15.01 TgC for *Pinus massoniana*, 11.51–13.44 TgC for *Cryptomeria fortunei*, 15.94–24.13 TgC for *Cunninghamia lanceolata*, 28.05 TgC for *Cupressus* spp., 5.32–15.63 TgC for *Populus deltoides* and 5.87–14.09 TgC for *Eucalyptus* spp. The carbon stocks in these seven species account for 36.8%–41.4% of the total carbon stocks in all GGP stands over the next 50 years.

## Introduction

Research on carbon sinks in forest ecosystems has become an imperative in response to global climate changes caused by the increased concentrations of greenhouse gases, primarily carbon dioxide, in the atmosphere. Studies have shown that forest regeneration is one of the major carbon sinks on land [1–3]. Landmass were converted from "carbon sources" to "carbon sinks" in the United States by the year 1945, primarily due to the transformation of abandoned farmland into forests through improving forest regeneration and the prevention of forest fires [4]. A similar conversion occurred in China in the 1980s and was attributed to large areas of forest regeneration [5].

The Grain for Green Program (GGP), established by the Chinese government, is considered one of the largest afforestation and reforestation projects worldwide. The program aimed to add more than 30 million hectares of plantation on cropland or barren land during its first strategic planning period (2001–2010) [6], which would significantly impact the carbon sink capacity of terrestrial ecosystems in China. Some previous studies have explored the carbon sequestration potential of GGP stands in China. Some of the studies deal with a few species at the county level [7–12] and others deal with only soil carbon at the inter-provincial/national level [13–17]. In addition, Chen et al. forecasted the carbon sequestration potential of GGP stands at the provincial level, but these results likely suffer from a larger error due to changes in the planted areas during the last 3 years [18]. Liu et al. [19] used process-based ecosystem modeling to assess the carbon sequestration potential of GGP stands only from cropland at the national level, but their model has a large uncertainty due to the omission of many factors. The GGP stands acreage in Southwest China (including Yunnan, Guizhou, Sichuan, Chongqing and Tibet provinces/city/region) accounts for 15% of the national total. Correspondingly, the GGP stands acreage and carbon sequestration capacity of each province, except Tibet, are in the top 10 of the 25 provinces with GGP stands in China [19]. In this paper, GGP stands, based on data from Southwest China, were selected as research areas to obtain reliable estimates of the carbon sequestration potential of GGP stands at the provincial and inter-provincial levels. This study aims to provide a resource for estimating the carbon sink potential of GGP stands across China and improving the forest management in this region.

## Materials and Methods

The institutions that granted permission are GGP Office in Forestry Department of Yunnan Province, GGP Management Center in Forestry Department of Guizhou Province, GGP Management Center in Forestry Department of Sichuan Province, GGP Office in Forestry Department of Tibet Autonomous Region and GGP Management Center in Forestry Department of Chongqing Municipality. The owners of the GGP land are like to cooperate with the provincial GGP office/management center for verifying the yearly planted areas for each tree species as this work is conducive to their compensation from the government. In addition, the field studies did not involve endangered or protected species.

### Data sources

Under the guidance and help of the provincial GGP office/management center the vast majority of afforestation tasks for the GGP were completed in Southwest China during the most recent strategic planning period, including the pilot phases in Sichuan during 1999–2001 and in Yunnan, Guizhou and Chongqing during 2000–2001. The implementation of the annual afforestation tasks had been verified by the provincial GGP office/management center with the assistance of the owners of the GGP land. The data of the yearly planted areas for each tree species under the GGP in this region during 1999–2010 were collected from the annual verification reports (2003–2011) of the GGP prepared by the provincial GGP office/management center in Yunnan, Guizhou, Sichuan, Chongqing and Tibet, respectively (Table 1 and S1 Table).

**Table 1:** Afforestation area and major tree species under the Grain for Green Program in Southwest China.

Province	Number of counties involved	Area(ha) ^a^	Major species (each species’ acreage accounts for 3.6%–10.7% of the total afforestation acreage under the GGP in Southwest China)	Data sources
Yunnan	126	822,778	*Pinus armandi*; *Cryptomeria fortune*; *Cunninghamia lanceolata*; *Cupressus* spp.; *Populus deltoides*; *Eucalyptus* spp.	The annual verification reports of the GGP prepared by Yunnan Forestry Department from 2003 to 2011(unpublished).
Guizhou	96	985,458	*Pinus armandi*; *Pinus massoniana*; *Cryptomeria fortune*; *Cunninghamia lanceolata*; *Cupressus* spp.; *Populus deltoides*; *Eucalyptus* spp.	The annual verification reports of the GGP prepared by Guizhou Forestry Department from 2003 to 2011 (unpublished).
Sichuan	142	1,523,891	*Pinus armandi*; *Pinus massoniana*; *Cryptomeria fortune*; *Cunninghamia lanceolata; Cupressus* spp.; *Populus deltoides*; *Eucalyptus* spp.	The annual verification reports of the GGP prepared by Sichuan Forestry Department from 2003 to 2011 (unpublished).
Chongqing	39	1,046,768	*Pinus massoniana*; *Cunninghamia lanceolata*; *Cupressus* spp.	The annual verification reports of the GGP prepared by Chongqing Forestry Department from 2003 to 2011 (unpublished).
Tibet	49	82,892	*Populus deltoides*	The annual verification reports of the GGP prepared by Tibet Forestry Department from 2003 to 2011 (unpublished).
Total	452	4,461,788	*Pinus armandi*; *Pinus massoniana*; *Cryptomeria fortune*; *Cunninghamia lanceolata*; *Cupressus* spp.; *Populus deltoides*; *Eucalyptus* spp.	

^a^ The number is the sum of yearly afforestation acreage during 1999–2010.

### Methods for assessing carbon sequestration potential

The changes in carbon stocks in the living tree biomass (including aboveground and belowground) and soil organic matter in the GGP stands were used to figure out the carbon sequestration potential of these stands. The dead organic matter (litter and dead wood) represents another type of biomass carbon pool in the GGP stands, which is usually accumulating but exhibits a lower rate of change compared to living tree biomass, as all of the studied stands were less than 60 years old. The carbon stocks of dead organic matter were therefore not included in this study in line with the conservative principles suggested in the *IPCC Good Practice Guidance for LULUCF* due to the lack of data and uncertainty in forecasting methodology [20].

#### Carbon stock and its changes in living tree biomass

The present study used the carbon stock change method developed by Chen et al. [18] to estimate the carbon stock changes in the living tree biomass of the GGP stands using the following formula (1):

$$CST_{i}=\sum_{j}\sum_{k}S_{jk}\times V_{ijk}\times WD_{j}\times BEF_{j}\times CF_{j}$$ (1)

where the meanings of each factor and its subscript are same as that in Chen et al. [18].

The *BEF*, *WD* and *CF* used in formula (1) were taken from the *Checklist report of land-use change and forestry greenhouse gas emission in China* (Table 2) [21].

**Table 2:** Values of wood density (*WD*), biomass expansion factor (*BEF*), carbon fraction (*CF*) and harvest rotation (*HR*) for different species.

Species/species groups	*WD*(Mg m^-3^)	*BEF*(whole tree)	*CF*(g g^-1^)	*HR* (yr)
*Larix gmelini*	0.490	1.74	0.51	61
*Pinus armandi*	0.396	2.29	0.50 ^a^	51
*Pinus yunnanensis*	0.483	2.04	0.54	51
*Pinus kesiya* var. *langbianensis*	0.454	1.83	0.50 ^a^	51
*Pinus massoniana*	0.380	1.80	0.54	51
*Pinus tabulaeformis*	0.360	1.96	0.50	51
*Abies* spp.	0.366	2.12	0.49	61
*Picea asperata*	0.342	2.12	0.51	101
*Keteleeria* spp.^b^	0.448	2.23	0.50 ^a^	51
*Metasequosia glyptostroboides*	0.278	1.90	0.50 ^a^	36
*Cryptomeria fortunei*	0.294	1.91	0.50 ^a^	36
*Cunninghamia lanceolata*	0.307	1.92	0.49	36
*Cupressus* spp.	0.478	2.11	0.50	101
Mixed conifer	0.405	2.00	0.52	69
*Cinnamomum camphora*	0.460	1.89	0.49	71
*Quercus* spp.	0.676	2.09	0.50	71
Hardwood broadleaf	0.598	2.34	0.50 ^a^	71
*Betula* spp.	0.541	1.62	0.50	51
*Sassafras tsumu*	0.477	2.49	0.50 ^a^	26
*Eucalyptus* spp.	0.578	1.65	0.50 ^a^	26
*Populus*.*deltoides*	0.378	2.16	0.51	26
*Vernicia fordii*	0.239	3.69	0.50 ^a^	26
Softwood broadleaf	0.443	2.50	0.50 ^a^	26
Mixed broadleaf	0.482	1.95	0.44	51

^a^ Generally used value.

^b^ Substituted by the value of similar species.

In this study, the empirical growth curves of plantation volume, developed based on the latest data from the National Forest Inventory (NFI) [22–25], were used for the GGP stands. The growth curve of stand volume for each species was expressed by formula (2):

$$V_{ijk}=a{\left(1-\text{exp}(-b\cdot A)\right)}^{c}$$ (2)

Where *A* represents stand age, *A* = *i-k*; and *a*, *b* and *c* are parameters to be fitted. The fitted growth curves, whose parameter values are included in Table 3, were used to estimate the stand volumes of the different species/species groups of the GGP stands in the target year *i*.

**Table 3:** Parameters of the fitted growth curves for the different tree species/species groups of plantation in Southwest China.

Province	Species/Species groups	a	b	c	R^2^	Samples
Yunnan	*Pinus armandi*	150.59	0.10	4.84	0.93	10
	*Pinus yunnanensis*	93.53	0.10	2.00	0.81	14
	*Pinus kesiya* var. *langbianensis*	101.38	0.17	2.43	0.54	20
	*Pinus* spp.	111.11	0.11	3.76	0.80	23
	Mixed conifer	100.35	0.11	3.76	0.60	42
	Softwood broadleaf	107.65	0.11	2.55	0.58	19
	Mixed broadleaf	59.51	0.08	1.89	0.64	13
	Mixed conifer and broadleaf	112.31	0.02	0.61	0.98	6
Guichou	*Pinus armandi*	92.52	0.07	2.77	0.83	12
	*Pinus massoniana*	122.74	0.05	1.16	0.83	11
	*Cunninghamia lanceolata*	226.84	0.03	1.12	0.85	19
	*Cupressus* spp.	304.22	0.01	1.67	0.64	7
	*Populus*.*deltoides*	163.53	0.08	3.66	0.93	9
	*Cryptomeria fortunei*	142.22	0.16	4.65	0.90	6
	Softwood broadleaf	161.92	0.05	1.33	0.76	12
	Mixed conifer	155.79	0.03	1.07	0.61	21
	Mixed broadleaf	62.11	0.27	6.21	0.69	6
	Mixed conifer and broadleaf	146.60	0.08	1.96	0.73	16
Sichuan	*Pinus armandi*	93.80	0.11	4.71	0.69	11
	*Pinus massoniana*	119.66	0.06	2.24	0.90	11
	*Pinus yunnanensis*	122.08	0.05	2.31	0.89	11
	*Cupressus* spp.	161.22	0.01	0.87	0.93	8
	*Cryptomeria fortunei*	210.35	0.11	3.09	0.97	9
	*Cunninghamia lanceolata*	166.67	0.03	1.35	0.84	11
	*Picea asperata and Abies* spp.	167.05	0.01	1.81	0.57	7
	Mixed conifer	129.77	0.20	19.77	0.92	9
	Softwood broadleaf	188.14	0.03	1.78	0.90	25
	Hardwood broadleaf	99.52	0.01	1.10	0.74	11
Chongqing	*Pinus massoniana*	108.78	0.05	1.70	0.89	12
	*Cupressus* spp.	91.49	0.02	1.06	0.93	9
	*Cunninghamia lanceolata*	229.16	0.02	1.24	0.89	10
	Mixed conifer and broadleaf	63.23	0.12	2.42	0.74	18
	Softwood broadleaf	67.27	0.11	2.46	0.51	19
	Hardwood broadleaf	114.22	0.02	0.95	0.51	10
Tebit	*Populus*.*deltoides*	209.47	0.05	2.50	0.66	11
	Softwood broadleaf	177.32	0.06	2.41	0.68	8

The empirical growth curves developed from the NFI data for each province were used in that province only, except the supplementary areas with a lack of NFI data for individual tree species in neighboring provinces.

Of the GGP stands established in Southwest China, 95% belong to Ecological Service Forests and are classified as noncommercial plantations (refer to the Annual Provincial Verification Reports on the GGP corresponding to each province in Southwest China). These plantations may be harvested according to customary rotation guidelines, as defined by the *Technical Regulations for Ecological Service Forest* [26,27]. However, it is possible that these GGP stands will never be harvested, because they are often planted in remote mountainous areas and serve as ecological shelters. Therefore, the following two scenarios were examined:

Scenario A: no harvesting;
Scenario B: logging at customary rotation periods.

For scenario B, it was assumed that all harvested biomass would decompose and be converted to carbon emissions immediately, all harvested lands would be replanted immediately without intensive soil preparation after harvesting, and that logging residues would be cleaned out [18]. The customary harvest rotation (HR) for each stand is shown in Table 2 [26].

### Carbon stock and its changes in soil organic matter

There is a potential carbon sink in the soil following long-term afforestation. However, the changes of soil organic carbon (SOC) storage after planting are affected significantly by land use/cover types before planting and other biophysical conditions and these changes are usually nonlinear over time [28–31].

The land used for the GGP in China, similar to the marginal agricultural land used for afforestation in the US [32], consists largely of degraded croplands/barrens with relatively little initial SOC storage. Intensive soil preparation is not allowed, and the original vegetation must be maintained to the greatest possible extent to prevent soil erosion [33,34]. Therefore, SOC likely increases rather than decreases following afforestation on GGP lands in Southwest China, which is consistent with Song et al. [16]. A similar finding was previously observed in the USA [35,36], although Paul et al. (2002) summarized an initial SOC loss after the afforestation of croplands based on global data [30]. As per Chen et al. (2009) [18], the changing factors of SOC stocks proposed by Niu and Duiker (2006) were used in the present estimation because no homothetic factors were found locally [32], as conducted using formula (3):

$$SOCC_{i}=\sum_{j}\sum_{k}r\times (i-k)\times S_{jk}$$ (3)

**Fig 1:**
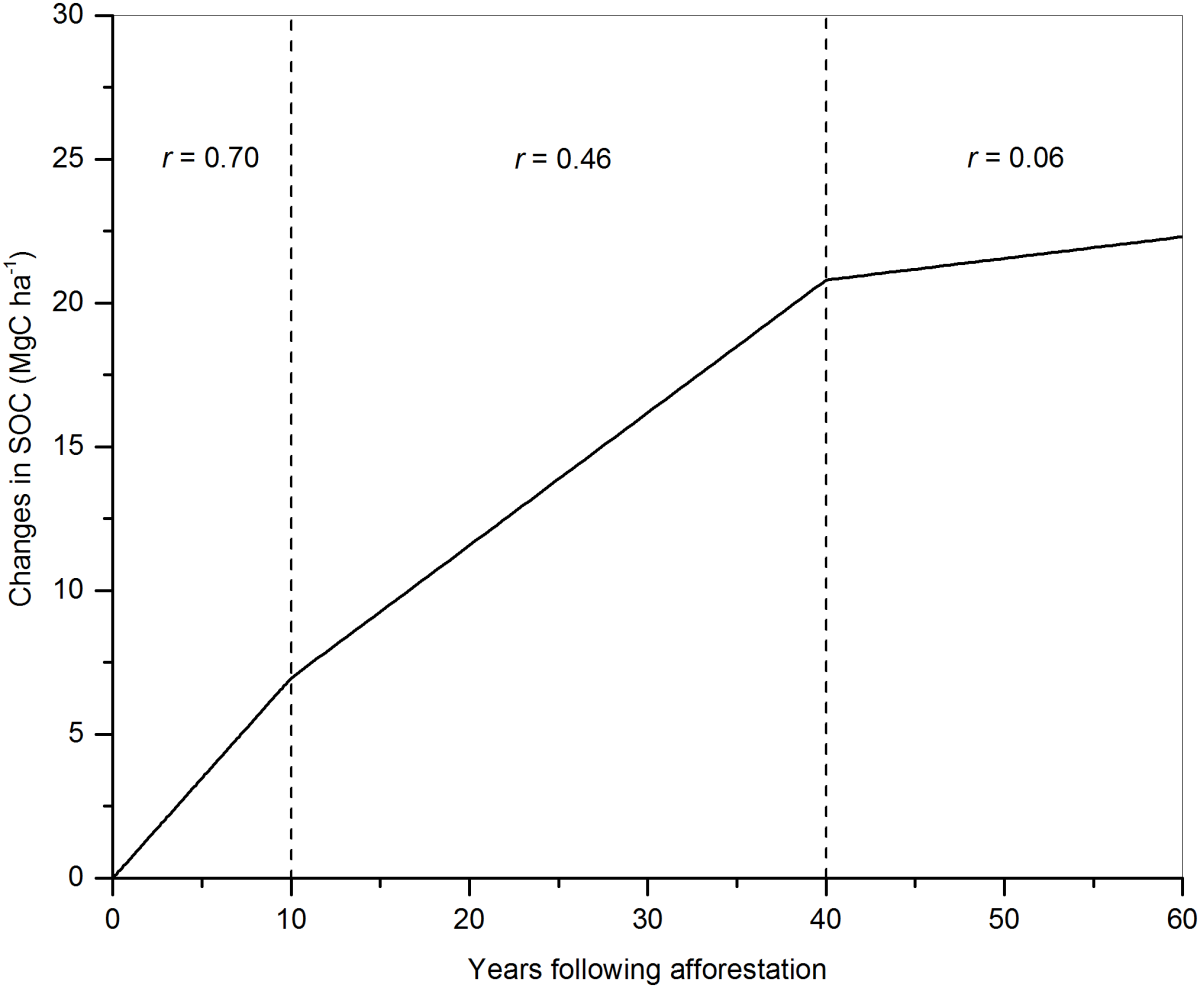
Change factors of soil organic carbon (SOC) over time. *r* represents the SOC change factor. Here the data came from the study of Niu and Duiker [24].

The values of *r* (Fig 1) result from the samples collected at 0–30 cm in depth, where the vast majority of SOC changes occur in plantations under the age of 60 years [30,31]. Some SOC changes may occur below 30 cm in these plantations but these were not included in the study based on the conservative principle and the lack of data.

## Results

### Future changes of carbon stocks in the GGP stands

**Fig 2:**
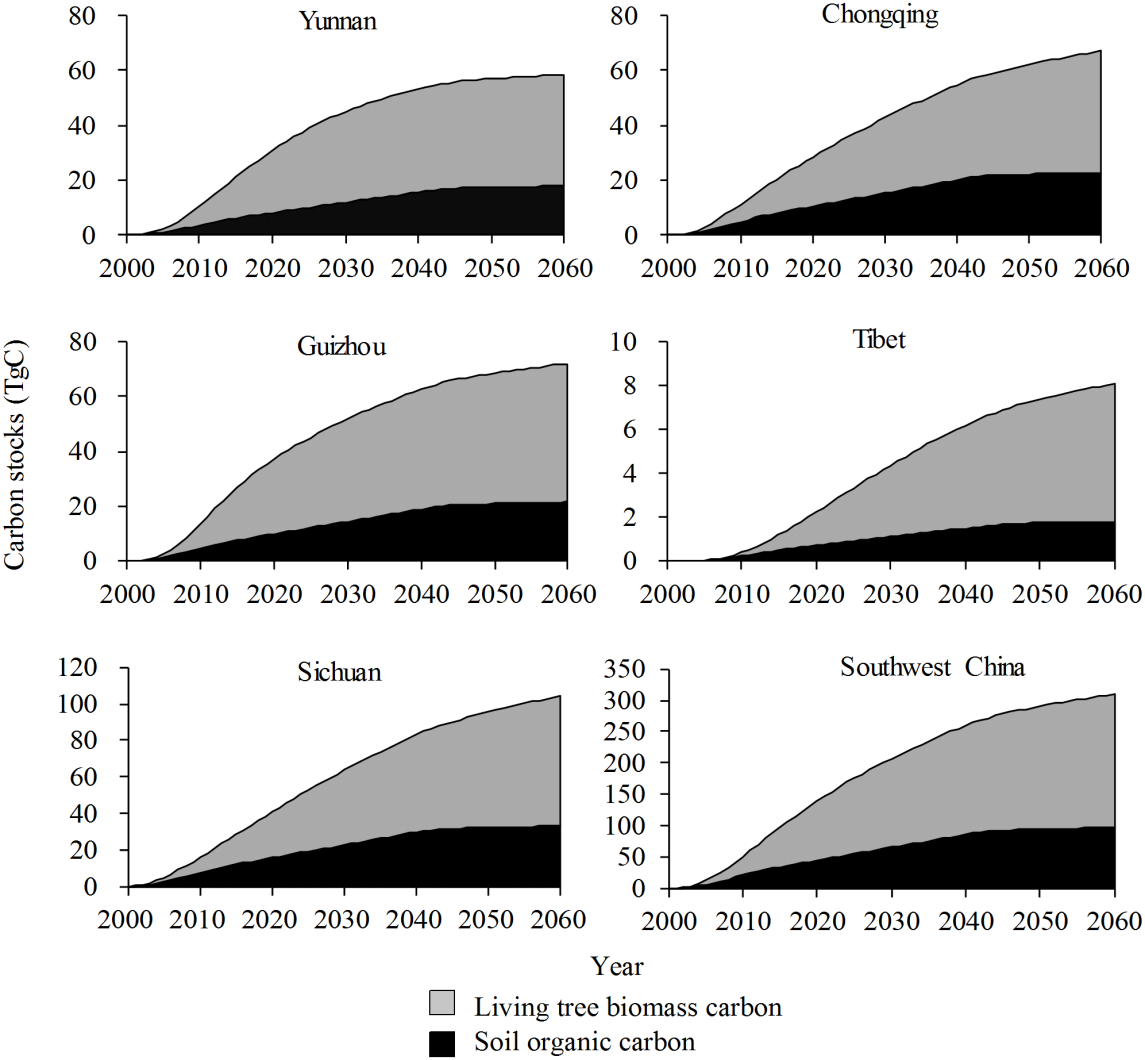
Changes of carbon stocks in stands under the Grain for Green Program under Scenario A in Southwest.

**Fig 3:**
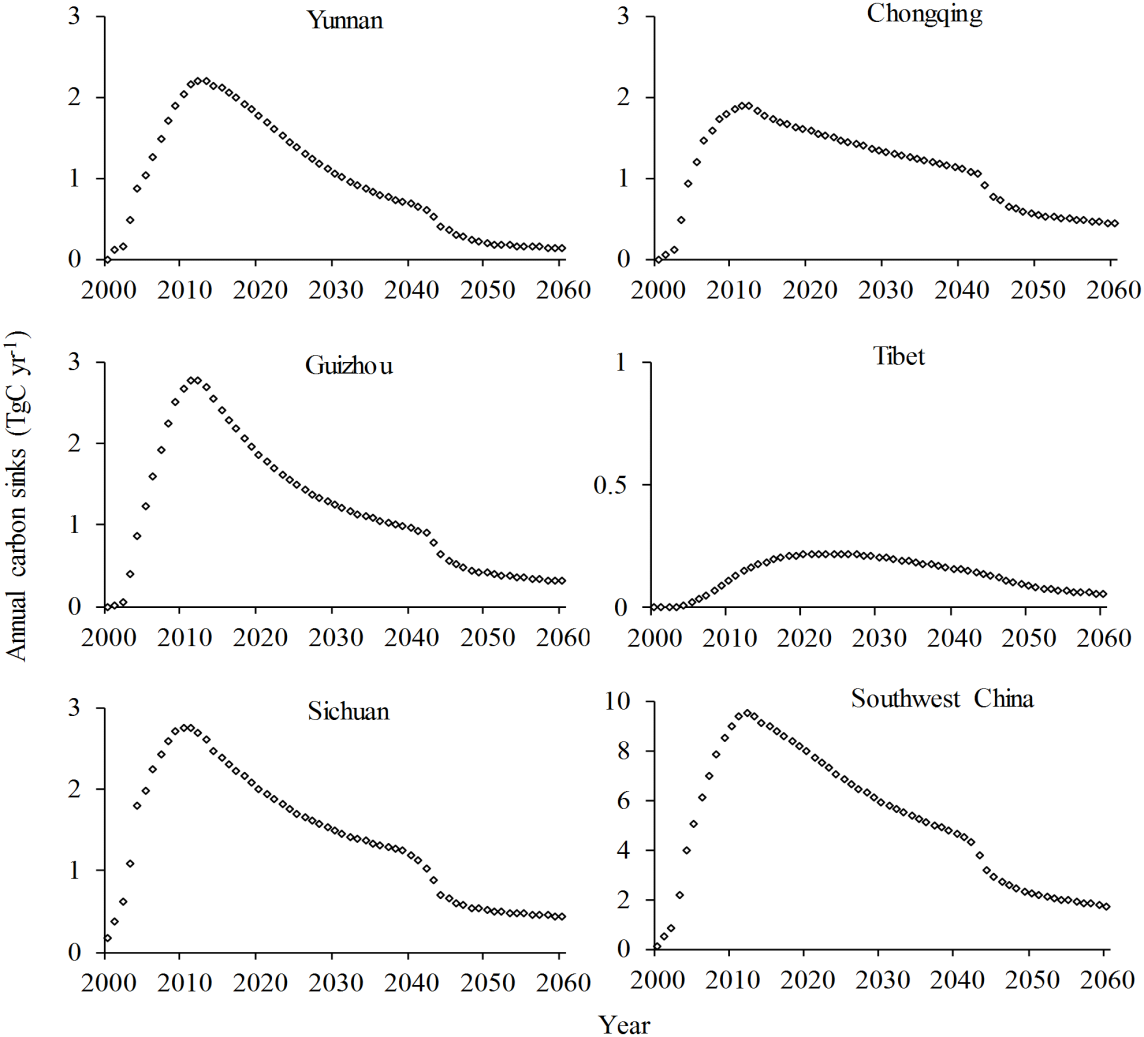
Changes of annual carbon stocks in stands under the Grain for Green Program under Scenario A in Southwest China.

Under Scenario A, the carbon storage in the GGP stands in Yunnan/Guizhou would rapidly increase until 2040, then slowly increase until 2060; storage in Chongqing would show a similar trend, but with a turning point in 2050; storage in Sichuan/Tibet would maintain its rapid increase until 2060; and the trend of carbon storage across all GGP stands in Southwest China would be similar to that in Yunnan/Guizhou (Fig 2). Under the same scenario, the changes of annual carbon storage in the GGP stands in Yunnan/Guizhou/Sichuan/Chongqing would peak in approximately 2012 and decrease sharply after 2040, but would not reach zero by 2060 except in Yunnan; changes in Tibet would peak at approximately 2025 and approach zero by 2060; and the changes of annual carbon stocks in all GGP stands across Southwest China would be similar to that in Sichuan/Chongqing, remaining an apparent yearly carbon sink until 2060 (Fig 3).

**Fig 4:**
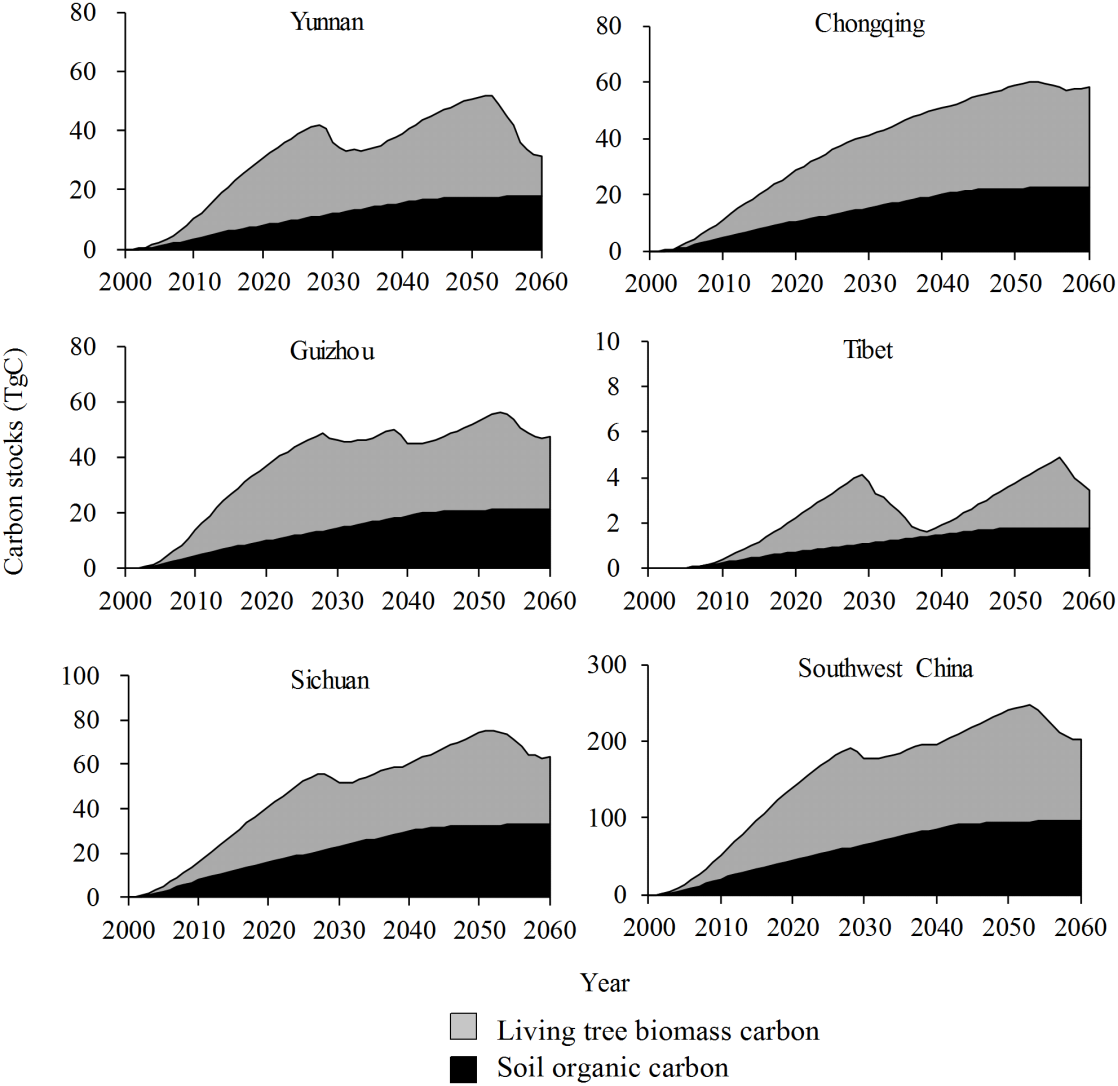
Changes of carbon stocks in stands under the Grain for Green Program under Scenario B in Southwest China.

**Fig 5:**
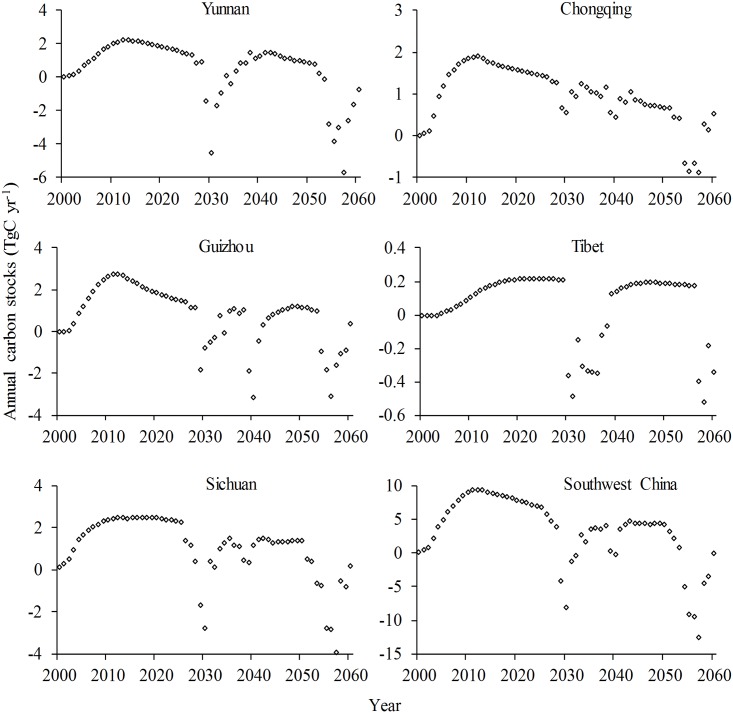
Changes of annual carbon stocks in stands under the Grain for Green Program under Scenario B in Southwest China.

Under Scenario B, the carbon storage in the GGP stands in Yunnan/Guizhou/Sichuan/Tibet would rapidly increase until 2027, then fluctuate from 2027 to 2060; storage in Chongqing would exhibit a steady increase until 2053, then fluctuate until 2060; and the trend of carbon storage across all GGP stands in Southwest China would be similar to that in Yunnan/Guizhou. The comparison of the amplitude of carbon storage changes under this scenario indicates that the largest changes would occur in Yunnan and Tibet, the smallest in Chongqing and moderate changes in Guizhou and Sichuan (Fig 4). Under the same scenario, the changes of annual carbon storage in the GGP stands in each province of Southwest China would reach their highest peak, after two to three fluctuations, before 2060; the peak for Yunnan/Guizhou/Sichuan/Chongqing would appear in 2012, while that of Tibet would emerge around 2025; negative annual carbon stocks would occur around 2030 and 2057 in Yunnan/Sichuan, around 2029, 2040 and 2056 in Guizhou, during 2030–2036 and around 2058 in Tibet and during 2054–2057 in Chongqing; and the changes of annual carbon storage in all GGP stands across Southwest China would be similar to that in Sichuan (Fig 5).

Carbon storage and its annual changes in the GGP stands in each province of Southwest China for particular years in the future under the two scenarios are shown in Table 4.

**Table 4:** Carbon stocks (CS) and annual carbon stocks (ACS) in stands under the Grain for Green Program in Southwest China.

Province	Scenario	Category	Year				
			2020	2030	2040	2050	2060
Yunnan	A	CS(TgC)	30.95	44.92	53.27	57.02	58.51
		ACS(TgCyr^-1^)	1.83	1.08	0.68	0.20	0.13
	B	CS(TgC)	30.95	35.99	38.95	50.64	31.22
		ACS(TgCyr^-1^)	1.83	-4.56	1.28	0.86	-0.73
Guizhou	A	CS(TgC)	36.97	50.72	62.40	68.48	71.95
		ACS(TgCyr^-1^)	1.86	1.24	0.96	0.41	0.31
	B	CS(TgC)	36.97	45.20	45.14	53.09	47.23
		ACS(TgCyr^-1^)	1.86	-0.80	-3.15	1.19	0.36
Sichuan	A	CS(TgC)	41.00	63.80	83.08	95.53	104.52
		ACS(TgCyr^-1^)	2.49	2.10	1.77	1.00	0.83
	B	CS(TgC)	41.00	51.45	60.21	74.30	63.27
		ACS(TgCyr^-1^)	2.49	-2.77	1.17	1.40	0.20
Chongqing	A	CS(TgC)	28.45	42.75	54.73	62.21	67.01
		ACS(TgCyr^-1^)	1.58	1.32	1.11	0.55	0.44
	B	CS(TgC)	28.45	41.08	50.64	58.66	58.08
		ACS(TgCyr^-1^)	1.58	0.54	0.45	0.68	0.53
Tibet	A	CS(TgC)	2.21	4.37	6.16	7.39	8.05
		ACS(TgCyr^-1)^	0.21	0.21	0.16	0.09	0.05
	B	CS(TgC)	2.21	3.80	1.91	3.77	3.42
		ACS(TgCyr^-1^)	0.21	-0.36	0.14	0.19	-0.34
Southwest China	A	CS(TgC)	139.58	207.55	259.65	290.62	310.03
		ACS(TgCyr^-1^)	7.96	5.95	4.67	2.24	1.75
	B	CS(TgC)	139.58	178.50	196.86	240.45	203.22
		ACS(TgCyr^-1^)	7.96	-7.95	-0.10	4.31	0.02

### Carbon sequestration functions in GGP stands of the major species

**Fig 6:**
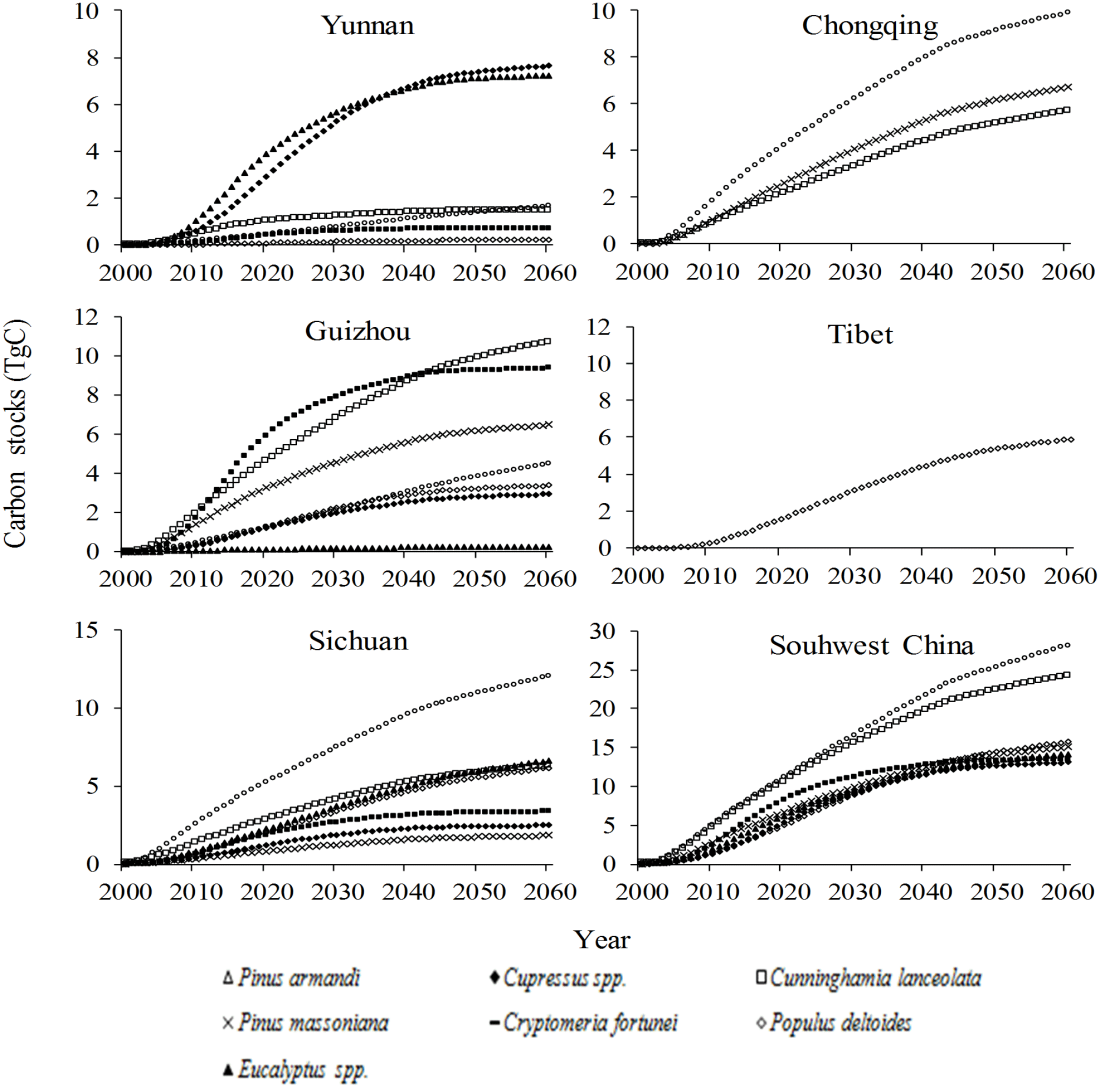
Changes of carbon stocks in every tree species of the stands under the Grain for Green Program under Scenario A in Southwest China.

In the recent strategic planning period for the GGP, seven tree species (i.e. *Pinus armandii*, *Pinus massoniana*, *Cupressus* spp., *Cryptomeria fortunei*, *Cunninghamia lanceolata*, *Populus deltoides* and *Eucalyptus* spp.) were planted on over 300,000 ha in Southwest China, accounting for 40.7% of all GGP stands in this region. All seven species were planted in Guizhou/Sichuan, while five were planted in Yunnan, three in Chongqing and one in Tibet. The tree species with the greatest carbon uptake potential in each region were *Pinus armandii* and *Eucalyptus* spp. in Yunnan, *Cryptomeria fortunei* and *Cunninghamia lanceolata* in Guizhou, *Cupressus* spp. in Sichuan/Chongqing and *Populus deltoides* in Tibet (Fig 6).

**Fig 7:**
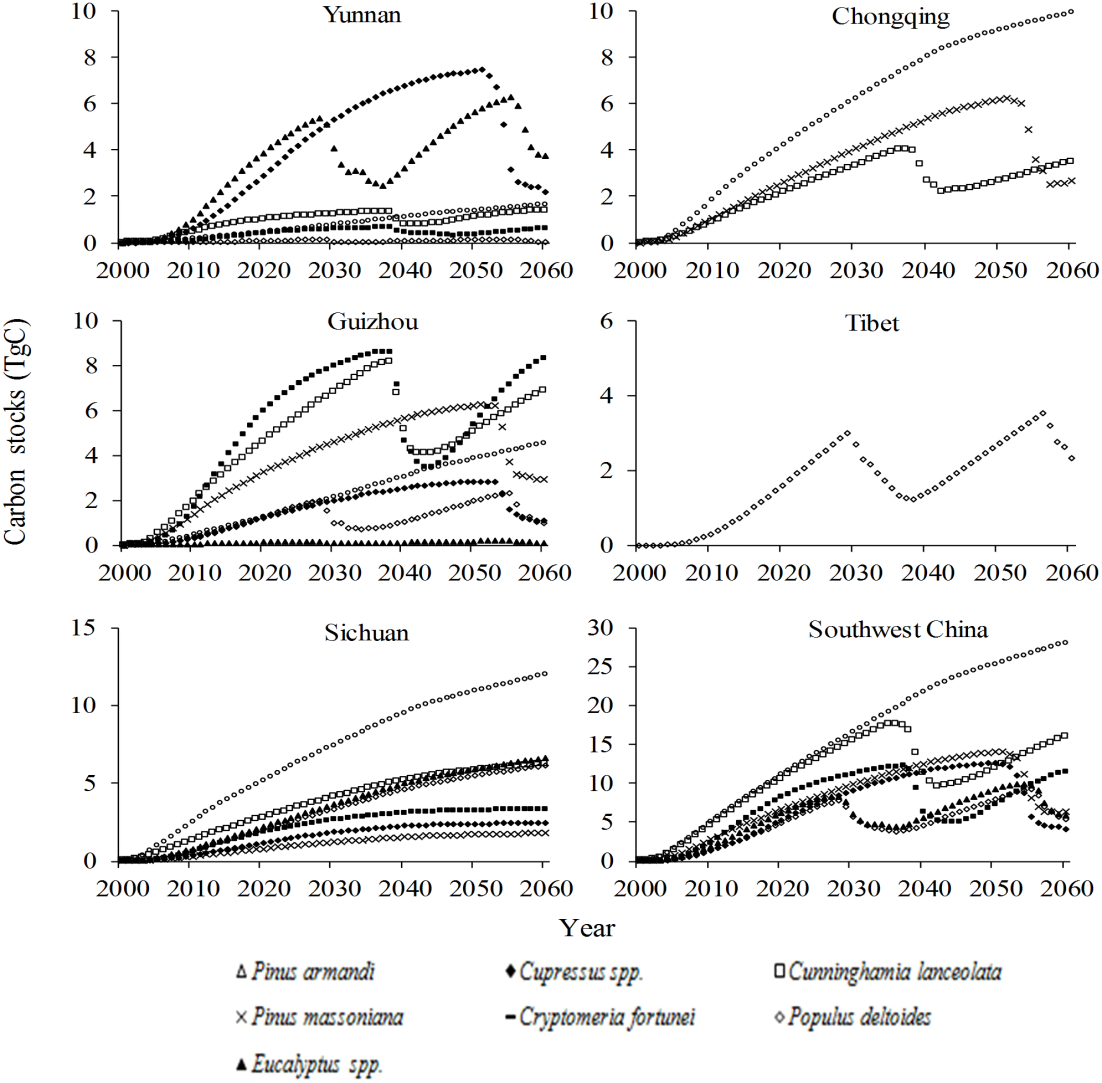
Changes of carbon stocks in every tree species of the stands under the Grain for Green Program under Scenario B in Southwest China.

Under Scenario B, carbon storage loss would occur in the stands of all these major species, except *Cupressus* spp. Losses are predicted to occur twice, around 2030 and 2057, in *Populus deltoides* and *Eucalyptus* spp. stands, but only once, around 2037, in stands of the other major species (Fig 7). The model estimates that the carbon stocks in the stands of these seven major species account for 37.9%–39.8% and 36.8%–41.4% of the stocks in all GGP stands under scenarios A and B, respectively, in Southwest China from 2020 to 2060.

## Discussion

### Contribution of the GGP stands to carbon stocks

Existing studies have shown that the total carbon storage in forest vegetation for each province of Southwestern China was 461.43 TgC for Yunnan [37], 175.50 TgC for Guizhou [38], 523.57 TgC for Sichuan, 443.98 TgC for Chongqing [39] and 329.64 TgC for Tibet [37] in the early years of this century. By 2060, the carbon stocks in the GGP stands under Scenario A are estimated to account for over 12.7%, 41.0%, 22.9%, 15.1% and 2.4% of the totals in Yunnan, Guizhou, Sichuan, Chongqing and Tibet, respectively. This projection indicates that the GGP stands will have different impacts on the carbon sink functioning of the forest ecosystems in each province over the next 50 years, being significant in Guizhou, moderate in Sichuan/Chongqing/Yunnan and minor in Tibet.

### Factors influencing carbon sequestration potential in the region

Based on the comparison of the expected results for 2060, the carbon sequestration potential in the GGP stands for the five provinces in Southwest China may be ordered as follows: Sichuan > Guizhou > Chongqing > Yunnan > Tibet. This result is approximately consistent with the acreages of the GGP stands, indicating that planted area is a main factor affecting the carbon uptake capability of the GGP stands in the provinces. Species is also an important factor affecting the carbon sequestration capacity in the stands. The carbon sequestration capacity of the GGP stands in Chongqing is not expected to surpass that of Yunnan until 2040, although the planted acreage in Chongqing is larger than that in Yunnan, because more fast-growing species are present in Yunnan than in Chongqing. The carbon sequestration potential of the GGP stands in Guizhou is larger than that in Chongqing, although Chongqing’s afforested area is slightly larger than that of Guizhou, because more tree species with larger volume are grown in Guizhou than in Chongqing. The maximum annual carbon sequestration of the GGP stands in Sichuan, Yunnan, Guizhou and Chongqing provinces was predicted to appear around 2012, while in Tibet, this point will be reached around 2024 due to the slow growth of the province’s alpine species. Forest management is also a key factor, because the carbon stocks and their annual increase in the GGP stands decrease with the implementation of logging.

### Uncertainties in carbon sequestration potential

Empirical growth curves are crucial to estimation models of carbon stocks in living tree biomass. The empirical growth curves directly affect the credibility of the estimation results. The stand volume density varies not only with tree species and age but also with site conditions and forest management. It is impossible to estimate the growth volume under specific site conditions and management practices due to a lack of the corresponding data and curves. However, the mean growth of plantations under a province’s diverse geographical environments and management practices can be characterized by the plantation growth curves fitted using the NFI data from this province, as reasonable sampling techniques were used for the NFI. These empirical growth curves may feasibly be used to estimate the mean growth volumes of GGP stands at the provincial level, as the GGP stands, like the previous plantations, are widely distributed in the mountains of the province [18]. In this paper, estimation models were established for each province/city/region in Southwestern China using the province’s own growth curves, except for a few individual tree species in which instances the growth curve from neighboring provinces were used. The estimation errors of carbon stocks arising from differences in environment conditions were therefore largely eliminated. In summary, these carbon estimation models used in this paper have a good credibility.

In addition to the accuracy of the estimation model, the determination of the credible potential carbon sequestration of an afforestation/reforestation project is often also dependent on the carbon baseline and leakage of the project [40–44]. The carbon baseline refers to the change of the carbon stocks within a project boundary in the absence of the project [41]. The carbon leakage refers to the measureable carbon emissions resulting from a project and occurring outside the boundaries of the project [44]. According to international rules, the carbon baseline and leakage must be deducted from the measured carbon sinks of a project. The Chinese GGP aims to restore forest vegetation on some croplands and barren mountainous lands. The choice of croplands for the GGP is restricted to decertified lands or those with a slope gradient of more than 25 degrees [33,34]. As shown in the research of Chen et al., these lands would continue to degrade, or at best remain in their current state, in the absence of the GGP [18]. The carbon baseline of the GGP stands can therefore be conservatively assumed as zero. The lands used for the GGP are often situated in remote mountainous areas, which are usually less populated. The Chinese government provides food and/or cash subsidies for farmers who have planted trees on their GGP-eligible croplands, which compensates these farmers for the loss of food due to the GGP [27]. Besides, the majority of GGP activity in Southwestern China is conducted manually instead of mechanically. These conditions have almost entirely eliminated the sources of carbon leakage, and the leakage of the GGP in this area may therefore be regarded as nil [18]. The carbon sequestration potential in the GGP stands is credible within the error range of the forecast models.

### Priorities for future research

Over a longer time scale than that employed in this study, the carbon stocks in dead organic matter should be included into the carbon sink potential of the stands, as the proportion of this component in the total carbon stocks of the stands will increase with the increased accumulation of litter and dead wood. Therefore, the carbon stocks in dead organic matter should be a major focus of further exploration.

In this study, we selected the SOC stock change factors proposed by Niu and Duiker (2006) to estimate the changes in SOC pool due to the limited observations on the SOC of GGP stands in Southwest China. The exact local soil conditions may differ from the reference case. Further studies should focus on the SOC changes in this region to precisely estimate carbon storage in the GGP stands.

In actuality, the majority of logged trees may be transformed into wood products with different life spans rather than decompose immediately. During the life spans of these wood products, it is reasonable to count their carbon storage, which has been reported to comprise 14.1%–32.0% of the total carbon stocks from logged trees [32,45]. The estimation of the carbon stocks in these harvested wood products, which were not included in this paper due to a lack of data, is another issue worthy of further exploration.

## Conclusions

The Chinese GGP is among the top afforestation and reforestation projects of this century to date. GGP stands have covered 4,973,103 ha with seven major forest tree species, with over 100,000 ha of this acreage in Southwest China, over the recent strategic planning period for the program. By the years 2020, 2030, 2040, 2050 and 2060, the expected carbon storages in the GGP stands planted during this most recent period are predicted to be 139.58 TgC, 207.55 TgC, 259.63 TgC, 290.62 TgC and 310.03 TgC, respectively, under conditions of no harvesting (Scenario A), and 139.58 TgC, 178.50 TgC, 196.86 TgC, 240.45 TgC and 203.22 TgC, respectively, under conditions of regular harvesting (Scenario B). For the same years, the estimated annual carbon stocks in these GGP stands are 7.96 TgC, 5.95 TgC, 4.67 TgC, 2.24 TgC and 1.75 TgC, respectively, under Scenario A, and 7.96 TgC, −7.95 TgC, −0.10 TgC, 4.31 TgC and −0.02 TgC, respectively, under Scenario B. In this region, the carbon storage in the seven major species of the GGP stands, which cover 47.5% of the area, account for 36.8%–41.4% of the storage in all species of the GGP stands over the next 50 years. The *Cupressus* spp., which possesses the largest acreage and the longest lifespan of the study species, plays a remarkable role in the carbon sink potential of the forests. By including the areas of five provinces and using the field verified data on the GGP stands and the new empirical growth curves of stands developed based on the latest data from the NFI, the current study provides more credible and useful results than those presented in the previous study [18]. Our results provide a useful basis for optimizing forest management in Southwest China.

## Supporting Information

S1 Table Dataset for the annual afforestation area(ha) of each tree species/stand under the Grain for Green Program in Southwest China. The Southwest China consists of Yunnan Province, Guizhou Province, Sichuan Province, Chongqing Municipality and Tibet Autonomous Region.
(XLSX)
